# Higher-order brain regions show shifts in structural covariance in adolescent marmosets

**DOI:** 10.1093/cercor/bhab470

**Published:** 2022-01-14

**Authors:** Shaun K L Quah, Lauren McIver, Edward T Bullmore, Angela C Roberts, Stephen J Sawiak

**Affiliations:** Behavioural and Clinical Neuroscience Institute, University of Cambridge, Cambridge, CB2 3EB, UK; Department of Physiology, Development and Neuroscience, University of Cambridge, Cambridge CB2 3EB, UK; Behavioural and Clinical Neuroscience Institute, University of Cambridge, Cambridge, CB2 3EB, UK; Department of Physiology, Development and Neuroscience, University of Cambridge, Cambridge CB2 3EB, UK; Behavioural and Clinical Neuroscience Institute, University of Cambridge, Cambridge, CB2 3EB, UK; Wolfson Brain Imaging Centre, University of Cambridge, Addenbrooke’s Hospital, Cambridge CB2 0QQ, UK; Department of Psychiatry, University of Cambridge, Cambridge CB2 0SZ, UK; Cambridgeshire & Peterborough NHS Foundation Trust, Cambridge CB21 5EF, UK; Behavioural and Clinical Neuroscience Institute, University of Cambridge, Cambridge, CB2 3EB, UK; Department of Physiology, Development and Neuroscience, University of Cambridge, Cambridge CB2 3EB, UK; Behavioural and Clinical Neuroscience Institute, University of Cambridge, Cambridge, CB2 3EB, UK; Department of Physiology, Development and Neuroscience, University of Cambridge, Cambridge CB2 3EB, UK; Wolfson Brain Imaging Centre, University of Cambridge, Addenbrooke’s Hospital, Cambridge CB2 0QQ, UK; Translational Neuroimaging Laboratory, University of Cambridge, Cambridge, CB2 3EB, UK

**Keywords:** adolescence, development, marmoset, network models, structural covariance

## Abstract

Substantial progress has been made studying morphological changes in brain regions during adolescence, but less is known of network-level changes in their relationship. Here, we compare covariance networks constructed from the correlation of morphometric volumes across 135 brain regions of marmoset monkeys in early adolescence and adulthood. Substantial shifts are identified in the topology of structural covariance networks in the prefrontal cortex (PFC) and temporal lobe. PFC regions become more structurally differentiated and segregated within their own local network, hypothesized to reflect increased specialization after maturation. In contrast, temporal regions show increased inter-hemispheric covariances that may underlie the establishment of distributed networks. Regionally selective coupling of structural and maturational covariance is revealed, with relatively weak coupling in transmodal association areas. The latter may be a consequence of continued maturation within adulthood, but also environmental factors, for example, family size, affecting brain morphology. Advancing our understanding of how morphological relationships within higher-order brain areas mature in adolescence deepens our knowledge of the developing brain’s organizing principles.

## Introduction

Adolescence is a period of transition from childhood to adulthood, marked by fundamental change in both anatomy and behavior. It is also a time of mental health vulnerability, covering the peak onset of many neuropsychiatric disorders, notably anxiety and mood disorders, psychosis and personality disorders (Kessler et al. 2005; Paus et al. 2008; Fuhrmann et al. 2015; Marín 2016). Indeed, a subset of adolescents displaying early pubertal maturation appear at increased risk of psychopathology (Graber 2013; Ullsperger and Nikolas 2017). But to understand such differences a far greater understanding of the timing of developmental change in the typical healthy brain is needed. Consequently, there have been a wealth of neuroimaging studies providing detailed trajectories of volumetric and functional changes throughout the brain across development in humans (Giedd et al. 1999; Gogtay et al. 2004; Blakemore 2008, 2012) and nonhuman primates (Malkova et al. 2006; Sawiak et al. 2018). Longitudinal MRI studies have demonstrated cortical thinning and thickening of gray matter, in addition to volumetric and microstructural changes within white matter, with the temporal and spatial patterning of these changes differing both across the brain and between individuals. Importantly, these maturational patterns map the major events of brain development occurring at the neuronal level in non-human primates (Zecevic et al. 1989; Bourgeois and Rakic 1993).

Network perspectives (Bullmore and Sporns 2009; Fornito et al. 2016) are increasingly used to extract salient information from these large neuroimaging datasets by considering the statistical associations of measures between different brain areas (Vértes et al. 2012). Initially these network perspectives focused on functional brain networks, derived from correlations between fMRI time series and axonal networks, created by reconstructing white matter tracts from diffusion tensor imaging. More recently, structural networks have also been established by calculating correlation coefficients between the macro-structural MRI metrics, for example, cortical thickness or regional volume of different brain regions (Alexander-Bloch, Raznahan, et al. 2013), or by estimating the morphometric similarity between macro- and micro-structural metrics measured at multiple regions (Seidlitz et al. 2018). Differences have been shown in the brain’s structural covariance networks for neuropsychiatric conditions including schizophrenia (Alexander-Bloch et al. 2014), obsessive–compulsive disorder (Yun et al. 2020), autism (Bethlehem et al. 2017), and post-traumatic stress disorder (Proessl et al. 2020). Although changes in structural covariance networks before adulthood have been studied in the human brain (Zielinski et al. 2010), structural covariance network changes from adolescence to adulthood such as that shown here have not. Recent findings have shown that maturational covariance (i.e., correlation between maturational trajectories of distinct brain regions) explains much of the observed structural covariance between regional brain volumes in adults (Alexander-Bloch, Raznahan, et al. 2013). Additional factors related to structural covariance networks include functional connectivity and transcriptomic similarity (Seeley et al. 2009; Alexander-Bloch, Giedd, et al. 2013; Yee et al. 2018).

Whilst such studies offer potential insight into the brain mechanisms that underlie normal as well as pathological developmental changes in behavior, nevertheless they cannot establish causality. Animal models thus have a crucial role in establishing brain-behavior relationships across development. Non-human primates are especially valuable since the structure and connectivity of their higher-order cortices involved in the regulation of cognition and emotion, that is dysfunctional in neuropsychiatric disorders, is more similar to humans than that of the more commonly used rodents (Kaiser and Feng 2015; Roberts 2020). However, before establishing such brain–behavior relationships it is important to first understand normative brain development in non-human primates and establish the comparability of brain development between humans and these model animals, especially in relation to the maturation of higher-order brain networks.

Using marmosets, we previously identified distinct maturational trajectories of regional volumes from infancy to adulthood (Sawiak et al. 2018) consistent with the delayed development of the frontal and temporal lobes in humans (Gogtay et al. 2004). Importantly, we revealed that the timing of structural maturation varied considerably between the multiple regions within prefrontal cortex. We hypothesized that such differences in maturational timing may result in distinct time windows of vulnerability within distinct prefrontal cognitive and emotion regulatory circuits, highly relevant to our understanding of the onset of neuropsychiatric symptoms. However, these findings were restricted to gray matter volume changes within individual brain regions and did not consider the complex interactions occurring between regions.

Here, we compare structural covariance brain networks in early adolescence (11.5–12.5 months, *n* = 31) and in adults (18–49 months, *n* = 31). We construct structural covariance networks using the morphometric volumes of 135 brain regions (Supplementary Table 1) measured via structural MRI at these early and late stages. First, we analyzed differences in the topological properties of these networks to provide insight into their changing dynamics, focusing on the late-developing frontal and temporal cortices. As the synchronized maturational trajectories of brain regions have been suggested to be an important predictor of structural covariance in humans (Alexander-Bloch, Raznahan, et al. 2013), we also tested whether a common organizational principle was shared between humans and nonhuman primates such that this relationship is also present in marmosets. To do this, we constructed maturational covariance matrices based on the brain volumetric trajectories of marmosets from infancy to adulthood (Sawiak et al. 2018). As this novel relationship between maturational covariance and adult structural covariance showed a gradient of declining strength from unimodal sensory areas to transmodal association areas (notably, the frontal pole and inferior temporal cortex), we explored factors that may underpin such a weakening of the relationship. Throughout the manuscript, to avoid any ambiguity with the term “connection” we distinguish associations between areas reflected by significant structural covariance as “graph connections” (i.e., “edges” in graph/network theory), in contrast to more literal (e.g., axonal, monosynaptic) connections wherever there is scope for confusion.

## Materials and Methods

### Animals

All procedures were conducted in accordance with the UK Animals (Scientific Procedures) Act 1986 under license PPL70/7618 and approved by the University of Cambridge Animal Welfare and Ethical Review Board. The cohort consists of 31 early adolescent (19 males; 12 females) marmosets scanned between 11 and 13 months of age (mean: 11.9 ± 0.3 months) and 31 young adult (17 males; 14 females) marmosets scanned after the age of 17 months (mean: 30.8 ± 7.9 months).

The adolescent cohorts were part of our previous developmental study (Sawiak et al. 2018) and at the time of imaging had not undergone any other experimental procedures. The adult animals, a distinct cohort from the same colony, had been screened with a single instance of the human intruder test (15 min) and model snake test (20 min) to assess their trait emotionality as part of the unit’s screening procedure. Structural MRI data from these animals have been previously reported in other studies (Mikheenko et al. 2015; Santangelo et al. 2016, 2019).

All animals were reared and housed under controlled temperature (24 °C) and humidity (55%) conditions, and were maintained on a 12-h light/dark schedule with light transition periods at dawn and dusk. Adolescent animals were housed in family groups, while adult animals were housed as male–female pairs (males were vasectomized). All animals were provided with a balanced diet and water ad libitum.

On the day of scanning, animals were fasted. Marmosets were sedated with ketamine (20 mg/kg; Vetalar solution 100 mg/mL Pfizer, Kent, UK) and intubated for isoflurane anesthesia (2.5% in 0.25–0.40 L/min O_2_). Throughout scanning, respiration rates and body temperatures were monitored. The isoflurane dose was adjusted between 1% and 3% to maintain respiratory rates in the normal range. After scanning, marmosets’ health was assessed regularly, including monthly weighing, as part of the normal colony procedure. One adult animal displayed difficulty breathing 12 days after scanning and was euthanized.

### Image Acquisition and Processing

Animals were scanned using a Bruker PharmaScan 47/16 MRI system (Bruker, Inc., Ettlingen, Germany) at a field strength of 4.7 T. A custom 6-cm birdcage coil was used for signal transmission and reception. Structural images were acquired using a rapid acquisition with relaxation enhancement (RARE) sequence (parameters: TR/TE_eff_ 11 750/23.5 ms, 125 slices of 250 μm thickness, echo train length 4 and 3 averages) in 21 m 44 s. The field of view was 64 × 50 mm yielding an isotropic resolution of 250 μm.

Images were processed using SPM8 (Wellcome Trust Center for Neuroimaging, UCL, UK) with the SPMMouse toolbox for animal data (Sawiak et al. 2013). Brains were aligned with tissue probability maps derived from marmosets from the same colony (Mikheenko et al. 2015) and segmented into gray matter, white matter, and cerebrospinal fluid (GM, WM, and CSF). DARTEL (Ashburner 2007) was used for image registration and to create population templates. Jacobian determinant maps for each scan were produced from the DARTEL flow fields.

### Structural Covariance Matrix and Graph Analysis

The volumes of 135 brain regions were estimated by integrating Jacobian determinants from the DARTEL transformations over each region mask, following Sawiak et al. (2018). A list for the parcellation of brain regions is given in Supplementary Table 1. The regions listed match those used in our previous study reporting developmental trajectories in the marmoset brain, and the construction of the atlas has been described previously (Sawiak et al. 2018). The atlas used labeled cortical regions based on cytoarchitecture determined histologically (Paxinos et al. 2012). Estimates of the uncertainty for the labels and justification for the use of the same cytoarchitectonic template for 12 month and adult animals is given in Supplementary Material with Supplementary Fig. 1, using probabilistic regional brain maps provided by Majka et al. (2021).

Correlation coefficients (Pearson’s *r*) between each pair of regions were calculated after correcting for the effects of sex, age, and TIV. Sex was included as a potentially explanatory factor for variation in regional volumes, although sex effects were not significant in regional growth trajectories in the developmental study (Sawiak et al. 2018). This was done separately for adolescent and adult cohorts to produce covariance matrices of each. To determine significant correlations, the false-discovery rate (FDR) was controlled for an adjusted *P* < 0.05. Two-tailed *P*-values are reported for significance.

After thresholding (*P*_FDR_ < 0.05) to control false-positive correlations between regional volumes, unsigned weights from the association matrices were used to construct graph models. Graph analysis was conducted in Matlab (r2019b, Mathworks, Inc.) with the brain connectivity toolbox, BCT (Rubinov and Sporns 2010). The community structure, representing a modular parcellation of the network was determined with Newman’s spectral algorithm for community detection (Newman 2013) as implemented in the BCT. Briefly, regions were subdivided into non-overlapping modules in a way that maximizes the number of graph connections within each module and minimizes the number of graph connections between modules.

#### Connection Distance

The mean Euclidean distance of connections of the network, $D(G)$ is defined as:

$$ D(G)=\frac{1}{m}\sum_{i\ne j}{A}_{ij}{d}_{ij} $$

where $m$ is the number of connections in the graph network, ${A}_{ij}=1$ if a connection links $i$ and $j$ and 0 if not, and ${d}_{ij}$ is the connection distance between two regions $i$ and $j$. This value was normalized to a range of 0 to 1 by dividing by the maximum mean connection distance of a network with the same number of connections (Alexander-Bloch, Raznahan, et al. 2013). 95% confidence intervals estimated for connection distances were calculated by bootstrapping, using the bias corrected and accelerated method for graph properties based on 2000 resamples with replacement for subjects. The connection density of the resampled networks was constrained to the connection density of the networks from the originaldata.

#### Clustering Coefficient

The clustering coefficient of an area, ${\tilde{C}}_i$ is the fraction of each area’s neighbors that are connected to each other by a single connection. The clustering coefficients are weighted by the covariance strength of connections, $w$ and given by:

$$ {\tilde{C}}_i=\frac{2}{k_i\left({k}_i-1\right)}\sum_{j,k}{\left(\tilde{w}_{ij}\tilde{w}_{jk}\tilde{w}_{ki}\right)}^{1/3} $$

where ${k}_i$ is the number of connections or degree of an area (Onnela et al. 2005). The weights are scaled by the largest weight of the area’s connections, $\tilde{w}_{ij}={w}_{ij}/\max ({w}_{ij})$. The clustering coefficient of each brain subdivision, $\tilde{C}(G)$ is the average of the clustering coefficients over all areas within each subdivision, $v$ defined as:

$$ \tilde{C}(G)=\frac{1}{V^{\prime }}\sum_{v\epsilon{V}^{\prime }}{\tilde{C}}_v $$

where $V^{\prime }$ is the number of areas with more than one connection.

### Maturational Covariance

Spline coefficients for regional volume and its first derivative as a function of age were calculated after correction for TIV and sex using the dataset of Sawiak et al. (2018). Spline knots covered 1.8–24 months spaced at 8-week intervals providing 13 volume and 13 derivative coefficients per region (further details are given in the Supplementary Materials, in particular Supplementary Fig. 2). In contrast to our previous study (Sawiak et al. 2018), where volumes were averaged bilaterally to give the mean volume of each structure from left and right hemispheres, each hemisphere was treated independently.

Pearson’s *r* was calculated between paired sets of 26 coefficients for each region and every other region to produce the maturational covariance matrix. Main effects of age and family size for each regional volume were assessed using a general linear model, which also included TIV and sex. Family size (range = 4–17) refers to the number of other individual animals (parents and siblings) in the home cage at the time of the 12-monthscan.

### Comparison with Tract-Tracing Data

To visualize the extent to which the structural covariance network reflects direct axonal connectivity between areas, we produced a matrix from an open-access resource of tracer injections in adult marmosets (Majka et al. 2020). These retrograde tracer data are directional (showing only intra-hemisphere connectivity) and the analysis of projecting regions was restricted to the cortex. Data from 143 injections assessed in 116 regions (combined from either left or right hemisphere injections) were summed into the 53 unilateral cortical regions used for the MRI analysis. The data available covered 35 of the 53 regions.

## Results

### Structural Covariance from Adolescence to Adulthood in the Marmoset Brain

**Figure 1:**
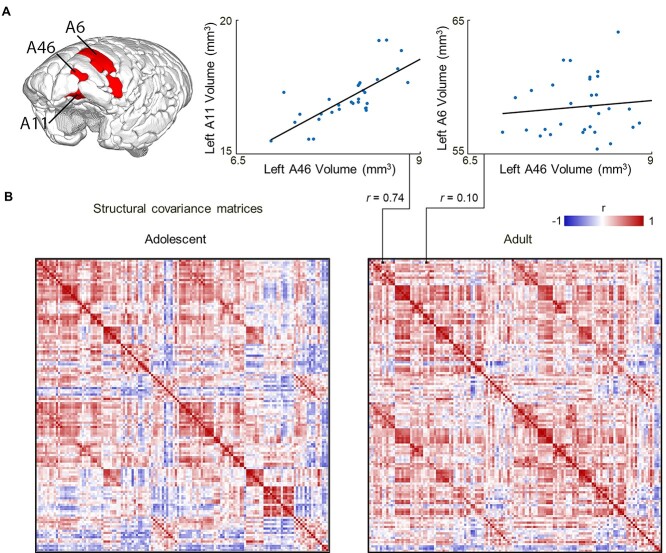
Structural covariance of adolescent and adult marmoset brains. (*A*) Illustration of the cortical parcellation of brain regions. Examples of high covariance (area 46 vs. area 11) and low covariance (area 46 vs. area 6) are shown for pairs of regions that are relatively equidistant. (*B*) Covariance matrices representing correlations between corrected regional volumes for all possible pairs of regions are shown for adult and adolescent animals. Red denotes positive covariance, blue indicates negative covariance, and white indicates near-zero covariance.

To characterize the relationship between the structural development of individual brain regions, we estimated the inter-regional correlation (Pearson’s *r*) of cortical volumes measured by structural MRI in 135 parcellated brain regions in 31 adolescent and 31 adult animals (Fig. 1*A*). Cortical volumes within each cohort were corrected for age, sex and total intracranial volume (TIV) before pair-wise correlations were estimated for all possible pairs of regions and compiled to form a structural covariance matrix for each group of images (Fig. 1*B*). Here, we see that pairs of regions separated by similar physical distance can have strong or weak covariance (Fig. 1*A*), and that the differences in structural covariance between pairs of regions varies considerably between the brains of adolescent and adult animals (Fig. 1*B*).

### Modular Re-organization of Structural Covariance for the Frontal and Temporal Cortices after Adolescence

**Figure 2:**
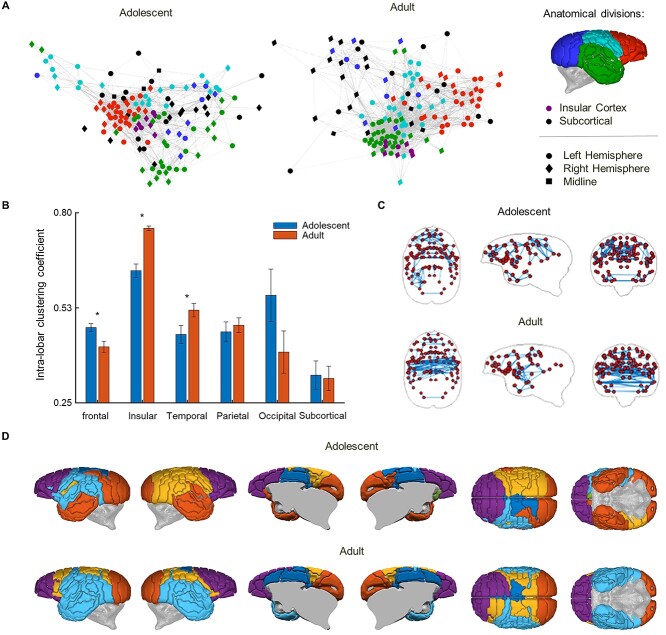
Structural covariance networks and modules in adolescent and adult marmosets. (*A*) Graphs of structural covariance networks for early adolescent and adult animals, with regional nodes colored according to major (lobar) subdivisions of brain anatomy and graph connections representing significant pair-wise correlations of regional volumes (*P*_FDR_ < 0.05). Closer spacing between connected nodes reflects greater graph connection weights (stronger correlations) between areas. The densest cluster within the structural covariance network shifts from regions of the frontal and insula cortex (orange and purple) during early adolescence, to regions within the temporal lobe and insula (green and purple) in adulthood. (*B*) Comparison of clustering coefficients for connections within each major (lobar) subdivision between adolescents and adults. Frontal, insular and temporal lobes showed significantly altered clustering coefficients after adolescence. Error bars represent standard error of the mean, SEM. (*C*) The strongest 2% of graph connections shown by anatomical (axial, sagittal and coronal) position. This schematic highlights the strongest connections in the brain and shows a clear shift of reduced frontal connections and increased temporal connections in the adult network relative to that of adolescents. (*D*) Modular decomposition demonstrated a community structure of six modules in both adolescent and adult structural covariance networks. The smallest module in both age groups was comprised of subcortical structures. A gray panel has been added in (*D*) for clarity, to obscure the internal surfaces of the cortex. The adult network community structure shows greater hemispheric symmetry compared to adolescents, particularly in the superior temporal lobes.

Weighted graphs representing brain networks of structural covariance were produced independently for adolescents and adults using a threshold calculated with the false-discovery rate controlled at *P*_FDR_ < 0.05. Regions of the frontal cortex show dense clustering in adolescence, reflecting high structural covariance between these regions (Fig. 2*A*). However, in adulthood, nodes representing regions of the frontal cortex become less clustered and the intra-lobar clustering coefficient (representing the average extent of clustering of structural correlations between regions within a lobe) of the frontal cortex decreases (*t*(62) = −2.86, *P* = 0.006) (Fig. 2*B*), reflecting a decrease in structural covariance and thus the number and strength of graph connections among regions of the frontal cortex. While this change partly reflects an increase in separation between granular prefrontal areas and non-granular (premotor) regions, there is also further separation within those prefrontal areas themselves. In contrast, regions of the temporal cortex transition from a distributed organization in adolescence to forming a dense cluster, alongside the insular cortex, in the adult brain (Fig. 2*A*). These regions show the opposite pattern to that observed in the frontal cortex, with intralobar clustering coefficients increasing in adulthood (temporal cortex: *t*(14) = 6.00, *P* < 0.001; insular cortex: *t*(59) = 2.17, *P* = 0.034) (Fig. 2*B*). The parietal and occipital cortex, and subcortical regions did not show significant changes (*P* > 0.05) in clustering coefficients with other regions within their own major subdivision (Fig. 2*B*) between adolescents and adults.

To further highlight age-related changes in the most strongly covarying regions, we visualized the adolescent and adult networks after thresholding to retain only the top 2% most positive (or negative) correlations between regional volumes. This demonstrated a dense pattern of strong graph connections in the frontal cortex in adolescence, contrasting with the emergence of strong inter-hemispheric graph connections in temporal cortex of adult animals (Fig. 2*C*) recapitulating the frontal to temporal shift observed in the overall network re-organization.

The modular organization of these networks was investigated using a spectral community detection algorithm (Newman 2013) applied separately to each structural covariance matrix. Networks for both age groups were composed of 6 modules of regions densely connected to other regions within the same module and sparsely connected to regions in different modules. The modules tended to comprise anatomically neighboring regions and mapped approximately onto the gross lobar divisions of the brain. The most marked age-related difference in modular organization was the formation of a single, bilateral temporal cortical module in the adult brain network in place of the two, hemispherically segregated modules of temporal cortical regions in the adolescent brain network. In short, the modular community structure of structural covariance networks became more symmetrical in the transition from adolescence to adulthood (Fig. 2*D*).

### Adult Brains have Greater Cross-Hemisphere Connectivity, PFC Segregation and Temporal Lobe Integration

**Figure 3:**
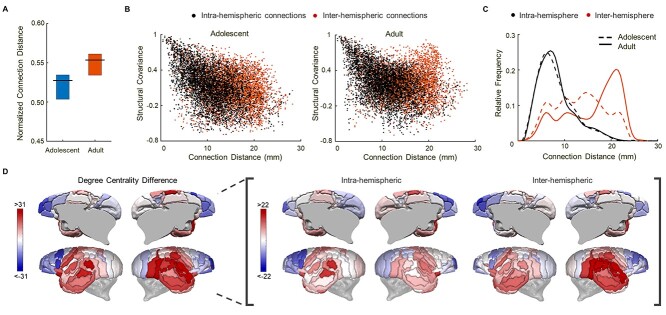
Age-related increase in long-distance inter-hemispheric graph connections, and changes in intra- and inter-hemispheric degree in adolescent versus adult marmoset structural covariance networks. (*A*) Distances of significant graph connections in adults tended to be higher compared to adolescents (*P* = 0.05). 95% confidence intervals from bootstrap resampling. (*B*) Pairwise structural covariance coefficients between all regions plotted against physical distance within (intra-: black) and between (inter-: orange) hemispheres in adolescent and adult marmosets. Generally, regions that are closer together show greater structural covariance as shown by the overall negative correlation between these measures. Exceptions to this were found in adults specifically with respect to some high distance inter-hemispheric connections (see high structural covariance cluster of orange on the right graph). (*C*) Relative frequency distribution of distance for significant intra- and inter-hemispheric connections within the structural covariance network for adult and adolescent animals. This confirms that while the frequency of intra-hemispheric connections according to distance did not differ between adolescents and adults the frequency of long range inter-hemispheric connections was markedly increased in adults. (*D*) Degree differences (change in the number of connections) between cortical regions of adults compared to adolescents. The most substantial changes between adolescent and adult networks included increases in degree in the inter-hemispheric connections of temporal regions (red) and reductions (blue) in intra-hemispheric connections laterally and inter-hemispheric connections medially in prefrontal regions.

The physical (Euclidean) distance corresponding to the strongest connections of the structural covariance network showed, on average, an increase in adults compared to early adolescents (Fig. 3*A*). Increasing numbers of strong inter-hemispheric graph connections across the midline in the adult network between bilateral homologous regions made a major contribution to this effect. Neighboring and more proximal regions tended to have higher structural covariance in both adolescents and adults (Fig. 3*B*). However, there was clearly an increased number of strongly connected, long distance, inter-hemispheric edges in the adult brain network. A comparison of the distributions of structural covariance with physical distance showed no evidence of a difference between adolescents and adults for connections within each hemisphere but strong evidence for changes in inter-hemispheric graph network connectivity in adults (Kolmogorov–Smirnov test: *D* = 0.03, *P* = 0.95; *D* = 0.29, *P* < 0.001 for intra- and inter-hemispheric connections, respectively; Fig. 3*C*).

Finally, to establish the relative importance of each region as a potential hub of the network, we measured the degree centrality (number of connections) of each region and the age-related change in degree or “hubness.” The most notable changes in degree centrality from early adolescence to adulthood were reductions within the prefrontal regions and increases within temporal lobe regions (Fig. 3*D*). The same finding was found considering both intra-hemispheric and inter-hemispheric degree although increases in the connectivity of temporal lobe regions were strongest for inter-hemispheric degree (Fig. 3*D*).

**Figure 4:**
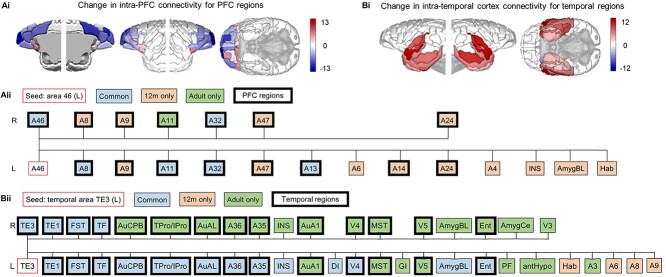
Age-related changes in intra-PFC and intra-temporal graph network connectivity. Detailed connections shown for left A46 and left TE3 as examples to illustrate the reduced intra-prefrontal and increased intra-temporal connectivity from adolescence to adulthood. Compared to adolescence, PFC regions in adulthood, overwhelmingly show a decrease in the number of other PFC regions with which they show significant covariance (*Ai*). Temporal regions show the opposite pattern with other temporal lobe regions (*Bi*). As illustrative examples of these general changes within lobes, graph connections of the left dorsolateral PFC, A46 (*Aii*) and the left temporal area, TE3 (*Bii*), with other brain regions in adolescence and adulthood are shown. Areas in red are connections that are lost after adolescence, areas in green are connections that are gained after adolescence, while areas in blue are connections that are retained after adolescence. Regions are sorted left-to-right in order of Euclidean distance from the seed region. Thick borders represent other regions within the same lobe of the seed region (PFC regions in *Aii*; temporal regions in *Bii*). Overall, left A46 showed reduced connectivity after adolescence, with the majority of connections lost with other PFC regions. In contrast, left TE3 increased in connectivity after adolescence, with new graph connections gained predominantly with contralateral temporal regions.

The reduction in frontal lobe connections within the structural covariance network was driven primarily by a loss of graph connections between PFC regions (Fig. 4*Ai*). Of 28 PFC regions spanning both hemispheres, 20 had fewer connections in adulthood. The eight regions to show an increase in frontal lobe connections included left A24, left A25, left A45, left proisocortical motor region (ProM), right A11, right A13, and bilateral orbital proisocortex (OPAI). Of these 20 regions with fewer connections in adulthood, all but one showed a reduction in connections with other PFC regions. As an example of specific regional connectivity difference in the adolescent to adult networks, the left dorsolateral prefrontal cortex (dlPFC, A46), a region important in the executive control functional network (Alves et al. 2019) covaries less with other nearby PFC regions (e.g., A47, A45, A24, A14, A9, A10) and the insula (DI, GI, INS), but gains graph connections with other regions critical for emotion regulation (e.g., A25, the OFC: A13 and OPAI, and the BLA) (Fig. 4*Aii*).

In contrast, temporal lobe regions showed increased connections mostly to other temporal cortical regions (Fig. 4*Bi*). Of 24 temporal regions, all but the amygdalopiriform transition area (APir) showed an increase in the number of connections, and all but the right piriform cortex of those included increased connectivity within temporal cortex. Comparison of connections lost and gained after adolescence within, for example, the left middle temporal gyrus (TE3), a representative region of the ventral attention network, revealed a small number of losses (ipsilateral A6, A8, A9, and habenula), a retention of connections with other ipsilateral temporal regions but substantial gains with contralateral (e.g., A35, A36, entorhinal cortex (ENT), MST, and auditory cortical regions: AuA1, AuCPB, and AuAL) and other ipsilateral temporal regions (e.g., AuA1, MST), visual association areas (V3, V4, V5), the insula (agranular, INS; granular, GI), and the amygdala (basolateral, BLA; central nuclei, CeA) (Fig. 4*Bii*).

### Maturational Covariance Couples Strongly to Structural Covariance, with the Exception of Rostral Prefrontal and Lateral Temporal Cortices

**Figure 5:**
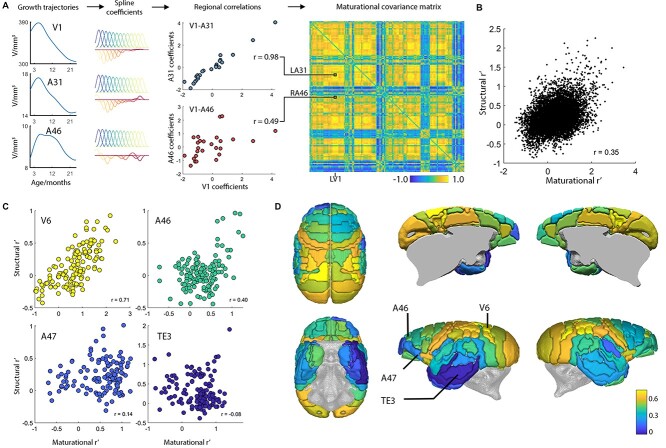
Regionally variable strength of coupling between adult structural covariance and maturational covariance. (*A*) Construction of the maturational covariance network. Spline coefficients from the growth trajectory of each region are compared with Pearson’s *r* to find regions with similar growth patterns. Exemplar regions primary visual cortex (V1) and dorsal posterior cingulate cortex (A31) show similar growth (with an early peak followed by steady volume reductions starting before puberty and continuing into adulthood). This contrasts with dlPFC (A46) which has a stable volume well into puberty with a later volume reduction. These differences are captured by the regional correlation plots of the spline coefficients. The *r* value for each pair of regions provides one entry in the maturational covariance matrix. (*B*) Comparison of maturational covariance with the adult structural covariance network, reveals a significant correlation (*P* < 10^−6^). Each point represents a pair of regions. (*C*) Region-wise comparisons of adult structural covariance and maturational covariance for exemplar regions to illustrate a strongly coupled relationship (V6), moderate coupling (A46), weak coupling (A47) and almost no relationship (TE3). (*D*) Cortical mapping of the degree of coupling between adult structural and maturational covariance (Pearson’s *r* as in (*C*)) for each region shows a caudal-rostral gradient with a tendency for higher-order association areas (particularly prefrontal and inferotemporal regions) to have a weaker relationship.

Previous studies in humans have shown that structural covariance in the adult brain is predicted in part by maturational co-ordination or synchronized maturation between areas of the brain (Alexander-Bloch, Raznahan, et al. 2013). To assess whether a similar relationship exists in marmosets, we created maturational covariance matrices by parameterizing non-linear trajectories of regional brain volume development, and the first derivatives of these trajectories (Sawiak et al. 2018). These maturational developmental trajectories were calculated after adjustment for TIV, sex, and age in common with the structural covariance matrices. Maturational covariance, calculated as the correlation between spline coefficients representing regional maturational developmental trajectories, was estimated for each pair of regions and standardized by Fisher’s *r*-to-*Z* transformation. We found that maturational covariance was significantly positively correlated with structural covariance in the adult marmoset brain network, similar to analogous results previously reported in humans (Alexander-Bloch, Raznahan, et al. 2013). This result indicates that on average, regions with stronger structural covariance had more similar maturational trajectories during adolescence and those with weaker structural covariance tended to differ in their growth trajectories (Fig. 5*A*,*B*). We repeated the calculation, region-wise, to see how the relationship between adult structural covariance and maturational covariance is maintained in different parts of the brain. We found striking differences between maturational and structural relationships, as exemplar regions of high (V6), middle (A46), and low (TE3) correspondence illustrate. There is a clear rostral-caudal gradient in the results showing a weakening of correspondence from the sensorimotor cortex to the frontal pole in addition to lateral parietal and inferotemporal regions (Fig. 5*C*,*D*). Intriguingly, the areas showing weaker correspondence between structural covariance and maturational covariance are “high-expanding” cortical areas in primate evolution (Hill et al. 2010; Sneve et al. 2019) shown to differ the most between primates of different brain sizes.

**Figure 6:**
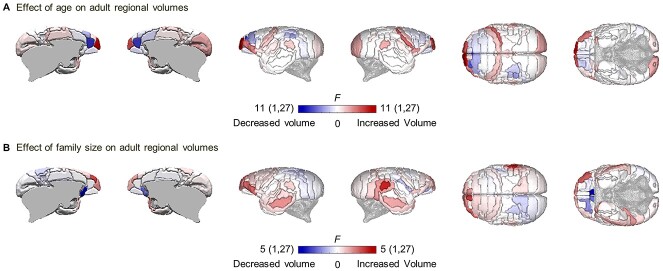
Areas showing the strongest effect of age and family size overlapped with areas showing the weakest coupling of maturational covariance with adult structural covariance. *F*-statistic main effects of age (*A*) and family size (*B*) on the regional volumes of adult animals with degrees of freedom. Regions showing an increase in volume associated with each factor in red, and regions showing a decrease in blue. Regions not affected by the factors are in white. Adult animal’s age ranged from 18 to 49 months. Family size is the total number of siblings and parents in the home-cage before animals leave the home-cage at ≈16 months old. The general linear model also included TIV and sex.

Building on this finding, we explored possible factors, which may explain why some regions may show weaker association between maturational covariance and adult structural covariance than others. This relationship is suggested to reflect coordinated neurodevelopment of brain morphology such that regions that grow in a similar pattern tend to covary in size when mature. Factors disrupting the coupling between maturational covariance and structural covariance include 1) further maturation outside of the 3–24-month window studied here and 2) differential environmental effects at an individual level impacting brain morphology. Either of these effects provide sources of variation on specific brain regions independently from the maturational covariance measures derived here. One such environmental effect, of relevance to those regions that showed a weaker association (namely rostral prefrontal and lateral temporal cortices) is social network size. The size of an animal’s social network as measured by family size has been shown to be positively associated with the morphology of these two regions of cortex in adult macaques (Sallet et al. 2011) and is a prominent environmental factor during development that varies markedly between individuals within a population. Thus, we estimated the effects of both adult age and family size on regional volumes within the adult cohort to determine whether the regions with the weakest relationship between maturational and adult structural covariance were also the regions in which volume was most affected by adult age (Fig. 6*A*) and/or family size (Fig. 6*B*).

In both instances, the strongest effects were found in the PFC including the rostral pole. In addition, family size, but not adult age, also showed a pronounced effect on regional volume in the lateral temporal cortex, in particular, area TE3 and right posterior parietal cortex. Thus, regions showing the strongest effect of these factors overlap with those regions showing the weakest relationship between maturational covariance and structural covariance. Among regions influenced notably by adult age, volume in the rostral PFC and other frontal regions were positively associated with this factor, while A32 within the medial PFC showed the opposite pattern. Consistent with the effect shown in macaques, greater family size was associated with expansion of the prefrontal and lateral temporal regions, though A25 within the medial PFC showed a shrinkage. The age effects shown here in the adult cohort (comprising marmosets aged from 18 to 49 months) reflect brain development continuing through adulthood, and could not have been seen in our previous study showing trajectories from infancy to adulthood (Sawiak et al. 2018) as it stopped at 27 months.

### Comparison of Structural Covariance with Axonal Connectivity

Comparison with tracer-defined axonal connectivity revealed that many of the stronger structural covariance connections corresponded with axonal connections (purple colored voxels in Supplementary Fig. 3). The considerable discrepancies, however, highlight that structural covariance cannot be explained as a straightforward consequence of direct axonal connectivity. Thus, as highlighted in the figure; in some cases, axonal connections are not reflected in structural connectivity (notably between the temporal lobes and orbital/medial prefrontal cortex; as well as between dorsolateral areas 45/47 and visual cortex), but also areas with significant structural covariance appear to have no direct axonal projections (e.g., areas of insular cortex with visual areas 4–6 or auditory cortex).

## Discussion

Although substantial progress has been made in determining morphological changes in specific brain structures during development, we have a more limited understanding of developmental effects on the inter-regional relationships of structural change, as defined by network analysis. By comparing the structural covariance brain networks of early adolescent and adult marmosets we reveal large-scale coordinated neurodevelopmental processes occurring during adolescence. In particular, we identify an apparent shift in the dominance of topological organization from the frontal to the temporal cortex. These are driven by reductions in the number and strength of prefrontal graph connections, in parallel with substantial increases in the number and strength of temporal graph connections, especially long-distance, inter-hemispheric connections between bilaterally symmetric areas of temporal cortex. Adult structural covariance was generally correlated with maturational covariance, meaning that regions that had strongly correlated volumes in the adult animals tended to share similar non-linear trajectories of development during adolescence. However, this relationship shows regional variation, with the strongest associations in sensory-motor areas while the weakest are found in fronto-temporal cortices, the latter regions influenced not only by continued maturation in early adulthood but also familysize.

In humans and rodents, brain regions that are closer together tend to covary more strongly in structural covariance (Alexander-Bloch, Raznahan, et al. 2013; Yee et al. 2018). We show here a similar tendency in both adolescent and adult marmosets. However, adults showed an increase in high structural covariance between distant graph connections. This transition from localized short-range graph connections early in life to distributed strong, long-range connections following maturation is consistent with patterns observed in functional resting state networks in humans (Power et al. 2010). Therefore, these changes in structural connectivity may be a useful indicator of the maturation of white matter tracts connecting distant brain regions during adolescence, particularly those primarily spanning hemispheres. However, it should be noted, and as shown here, increases in graph connectivity do not always depend upon axonal connectivity.

### PFC Segregation

Analysis of network topology revealed that frontal and insular regions formed a dense cluster and a large proportion of the strongest connections in the structural covariance network of adolescents. In contrast, frontal regions shifted to a more distributed topological arrangement in adulthood as shown by a reduction in their clustering coefficient with other frontal regions, likely reflecting a segregation of PFC regions into distinct, independent association and executive control networks. For example, there is a specific, localized prefrontal component in many of the resting state networks described in adults, including fronto-parietal executive, default mode and salience networks (Sylvester et al. 2012; Lopez et al. 2020). Consistent with this interpretation, neighboring motor areas of the frontal cortex did not show such a reduction in network connectivity as measured by the number of graph connections. At a cellular level, a rostral-caudal gradient in maturation has been previously shown in the marmoset frontal cortex, with more rostral prefrontal areas maturing later than the caudal motor/pre-motor areas (Burman et al. 2007) which may explain their later differentiation in adults shownhere.

This reduction in structural covariance connections and overall reorganization of PFC regions appears to reflect a shift from common factors governing PFC structural development at the start of adolescence to independent factors, likely related to specialized processing modules, by adulthood. The effects described for the dlPFC are similar to those seen across much of the PFC, suggesting that the PFC becomes more morphologically segregated from itself. Such changes may correspond to the ongoing development of association and projection tracts (Asato et al. 2010) shifting the connectivity of prefrontal regions from their anatomical neighbors to more specialized functional regions, leading to the matured transmodal functional networks present in adults (Fair et al. 2009; Buckner and Margulies 2019). Exceptions are primarily agranular regions of OFC, including caudal OFC, and subcallosal cingulate A25, which either show no change or an increase in PFC connectivity in adulthood. Consistent with our overall findings here, the greatest reduction in structural covariance connections in humans during adolescence are concentrated in frontal association regions (Váša et al. 2018); this reduction in structural covariance being linked to greater intracortical myelination during adolescence (Váša et al. 2018). This finding supports our interpretation that the divergence in morphology between prefrontal regions shown here reflects tuning of PFC white matter circuitry during adolescence by a combination of pruning local axonal connections based on anatomical proximity and consolidating longer-range axonal connections via myelination.

### Temporal Lobe Integration

In contrast to the PFC, temporal lobe regions displayed the opposite pattern. While there was a relatively distributed network in early adolescence, this developed into a dense cluster and formed the strongest connections in the structural covariance network in adulthood. Detailed analysis of the number of structurally covarying connections after adolescence in temporal regions showed that these increases were primarily mediated by marked increases in inter-hemispheric graph connections. This increase in contralateral connections may explain the establishment of symmetrical lateral, temporo-parietal junction modules representing regions in the dorsal and ventral attention network, the default mode network, and semantic network (Yeo et al. 2011) seen in adult but not adolescent humans (Schneiderman et al. 2007). The only marked increases in ipsilateral connections were within the fundus of the superior temporal cortex, MT, as well as the piriform cortex. The emergence of increased synchronized morphology across temporal regions of adult animals shown within this network may be driven by the development of contralateral white matter tracts and the continued development of sensory association networks within these regions in adolescence (Keshavan et al. 2002). Direct assessment of changing white matter tracts through development is ongoing as part of a follow-up longitudinal study including myelin mapping and diffusion tensor imaging, which should provide data to better address the underlying mechanisms of the changes we reporthere.

### Weak Maturational-Structural Coupling in Transmodal Association Areas

Research in humans suggests that the maturational development of brain regions plays an important role in the structural covariance networks seen in the adult brain. Specifically, regions with similar growth patterns across late childhood and adolescence show the highest levels of structural covariance in adults (Alexander-Bloch, Raznahan, et al. 2013). Beyond establishing the same relationship in marmosets, we also reveal that the strength of maturational-structural coupling shows a caudal to rostral gradient in regional variation. Hot spots where this coupling is strongest include unimodal sensory regions extending from the visual cortex to the somatomotor cortex. Cold spots, on the other hand, with the least-coupled regions peaked within the frontal pole and lateral and inferotemporal cortices; higher-order transmodal association areas corresponding to regions of the default mode network (Margulies et al. 2016; Buckner and Margulies 2019) and implicated in a social network circuit (Sallet et al. 2011). These regional patterns largely overlap with those shown in humans, though visual regions show weak coupling in humans but not here (Alexander-Bloch, Raznahan, et al. 2013).

We hypothesize that environmental factors, which have a marked effect on an individual’s brain morphology may contribute to the weak relationship between maturational development networks and adult structural covariance networks that are population-based. In the context of a lab-based marmoset breeding colony, animals develop within a highly controlled environment, which reduces inter-individual variation in their personal experiences. One remaining major source of such variation is the social environment, which is dominated by the size and compliment of their family unit. The finding therefore that family size during development had a significant effect on adult brain morphology in overlapping regions to those displaying a weak relationship between maturational covariance and adult structural covariance, for example, the frontal pole, is consistent with this hypothesis; although future studies would be required to determine any causal interaction. The other factor that may contribute to the weak maturational-structural coupling was continuing structural development across early adulthood. Indeed, morphology in the frontal pole showed such age-related associations.

## Conclusion

In summary, we reveal novel developmental changes in the structural covariance networks of higher-order brain regions across adolescence in the marmoset monkey. These findings extend and expand previous work suggesting that the structural covariance networks in the brain show distinct developmental patterns before and after adulthood. We not only demonstrate that organizing principles of the brain’s structural covariance network are generalized to nonhuman primates, but also identify regional patterns underlying these principles that shift across development. Future mapping of white matter tracts and their myelination across development will provide greater insight into the mechanisms that underlie the changes we report here. Moreover, extending these findings from a cross-sectional to longitudinal study will be important for validation, revealing individual differences in this profile, and relating these differences to cognitive and emotional development. Our findings not only advance understanding of how the brain matures during a critical window of development, but are also a crucial prerequisite for subsequent intervention studies using marmosets to study how alterations in the morphological relationships between brain regions may help predict vulnerability to neuropsychiatric disorders during development.

## Supplementary Material

Supplementary_bhab470

## Notes

We thank Yoshiro Shiba, Gemma Cockcroft, and Lydia Oikonomidis for help with performing the scans for these animals. *Conflict of Interest*: None declared.
