# Supplementary material for: Higher-order brain regions show shifts in structural covariance in adolescent marmosets
Source: Cereb Cortex. 2022 Jan 14;32(18):4128–40. doi: 10.1093/cercor/bhab470 (PMC9476623; doi:10.1093/cercor/bhab470)
Supplement: Supplementary_bhab470 [file supplementary_bhab470.docx]

Supplementary Information for

**Higher-order Brain Regions Show Shifts in Structural Covariance in Adolescent Marmosets**

Shaun K.L. Quah*^1,2^, Lauren McIver^1,2^, Edward T. Bullmore^1,3,4,5^, Angela C. Roberts^1,2^,
Stephen J. Sawiak*^1,3^

Corresponding Authors: Shaun K.L. Quah, Stephen J. Sawiak

Email: sklq2@cam.ac.uk, sjs80@cam.ac.uk

Contents

1: Structures parcellated in the template

2: Estimates of variability within cytoarchitectonic areas

3: Construction of maturational trajectory splines

4: Tract-tracing data vs. adult structural covariance networks

1. Supplementary Table 1: List of parcellated brain regions sorted by brain subdivision. Left and right regions were included separately in our analysis for bilateral brain regions (1-66).

|  | Brain Region | Abbreviation | Brain Region Subdivision | Hemisphere |
| --- | --- | --- | --- | --- |
| 1 | Area 10 | A10 | Frontal Pole | Bilateral |
| 2 | Area 11 | A11 | Orbitofrontal Cortex | Bilateral |
| 3 | Area 13 | A13 | Orbitofrontal Cortex | Bilateral |
| 4 | Orbital proisocortex | OPAl | Orbitofrontal Cortex | Bilateral |
| 5 | Area 46 | A46 | Dorsolateral Prefrontal Cortex | Bilateral |
| 6 | Area 9 | A9 | Dorsolateral Prefrontal Cortex | Bilateral |
| 7 | Area 14 | A14 | Ventromedial PFC | Bilateral |
| 8 | Area 25 | A25 | Ventromedial PFC | Bilateral |
| 9 | Area 32 | A32 | Ventromedial PFC | Bilateral |
| 10 | Area 45 | A45 | Ventrolateral Prefrontal Cortex | Bilateral |
| 11 | Area 47 | A47 | Ventrolateral Prefrontal Cortex | Bilateral |
| 12 | Proisocortical motor region | ProM | Ventrolateral Prefrontal Cortex | Bilateral |
| 13 | Agranular insular cortex | INS | Insular Cortex | Bilateral |
| 14 | Dysgranular insular cortex | DI | Insular Cortex | Bilateral |
| 15 | Granular insular cortex | GI | Insular Cortex | Bilateral |
| 16 | Proisocortex | TPro/IPro | Insular Cortex | Bilateral |
| 17 | Auditory cortex: primary area | AuA1 | Auditory Cortex | Bilateral |
| 18 | Auditory cortex: belt | AuAL | Auditory Cortex | Bilateral |
| 19 | Auditory cortex: parabelt | AuCPB | Auditory Cortex | Bilateral |
| 20 | Area 24 | A24 | Anterior Cingulate Cortex | Bilateral |
| 21 | Area 23 | A23 | Posterior Cingulate Cortex | Bilateral |
| 22 | Area 29 | A29 | Posterior Cingulate Cortex | Bilateral |
| 23 | Area 30 | A30 | Posterior Cingulate Cortex | Bilateral |
| 24 | Area 31 | A31 | Posterior Cingulate Cortex | Bilateral |
| 25 | Parietal area, PG: medial part | PGM | Posterior Cingulate Cortex | Bilateral |
| 26 | Area 4 | A4 | Motor and Premotor Cortical Regions | Bilateral |
| 27 | Area 6 | A6 | Motor and Premotor Cortical Regions | Bilateral |
| 28 | Area 8 | A8 | Motor and Premotor Cortical Regions | Bilateral |
| 29 | Area 1 | A1-2 | Somatosensory Cortex | Bilateral |
| 30 | Area 3 | A3 | Somatosensory Cortex | Bilateral |
| 31 | Secondary somatosensory | S2E | Somatosensory Cortex | Bilateral |
| 32 | Area 35 | A35 | Ventral Areas of the Temporal | Bilateral |
| 33 | Area 36 | A36 | Ventral Areas of the Temporal | Bilateral |
| 34 | Entorhinal cortex | Ent | Ventral Areas of the Temporal | Bilateral |
| 35 | Ventral temporal lobe | TF | Ventral Areas of the Temporal | Bilateral |
| 36 | Temporal area, TE1/2 | TE1 | Lateral and Inferior Temporal Cortical | Bilateral |
| 37 | Temporal area, TE3 | TE3 | Lateral and Inferior Temporal Cortical | Bilateral |
| 38 | Amygdalopiriform transition area | APir | Piriform Cortex | Bilateral |
| 39 | Piriform cortex | Pir | Piriform Cortex | Bilateral |
| 40 | Anterior intraparietal area | IP | Posterior Parietal Cortex | Bilateral |
| 41 | Occipito-parietal transitional area of cortex | OPt | Posterior Parietal Cortex | Bilateral |
| 42 | Parietal area, PE | PE | Posterior Parietal Cortex | Bilateral |
| 43 | Parietal area, PF | PF | Posterior Parietal Cortex | Bilateral |
| 44 | Parietal area, PG | PG | Posterior Parietal Cortex | Bilateral |
| 45 | Area 19 | A19 | Visual Cortex | Bilateral |
| 46 | Fundus of superior temporal sulcus | FST | Visual Cortex | Bilateral |
| 47 | Medial superior temporal area | MST | Visual Cortex | Bilateral |
| 48 | Visual area 1 | V1 | Visual Cortex | Bilateral |
| 49 | Visual area 2 | V2 | Visual Cortex | Bilateral |
| 50 | Visual area 3 | V3 | Visual Cortex | Bilateral |
| 51 | Visual area 4 | V4 | Visual Cortex | Bilateral |
| 52 | Visual area 5 | V5 | Visual Cortex | Bilateral |
| 53 | Visual area 6 | V6 | Visual Cortex | Bilateral |
|  | Subcortex |  |  |  |
| 54 | Medial dorsal thalamus | MDThal | Thalamus | Bilateral |
| 55 | Dorsolateral caudate nucleus | DLCaud | Striatal | Bilateral |
| 56 | Putamen | Put | Striatal | Bilateral |
| 57 | Ventromedial caudate | VMCaud | Striatal | Bilateral |
| 58 | Nucleus accumbens | Acb | Striatal | Bilateral |
| 59 | Caudate body | CaudBody | Striatal | Bilateral |
| 60 | Habenula | Hab | Subcortical Limbic | Bilateral |
| 61 | Anterior hypothalamus | antHypo | Subcortical Limbic | Bilateral |
| 62 | Lateral septum | LSeptum | Subcortical Limbic | Bilateral |
| 63 | Bed nucleus of the stria terminalis | BNST | Subcortical Limbic | Bilateral |
| 64 | Central nucleus of the amygdala | AmygCe | Amygdala-hippocampal | Bilateral |
| 65 | Basolateral amygdala | AmygBL | Amygdala-hippocampus | Bilateral |
| 66 | Anterior hippocampus | antHIPP | Amygdala-hippocampus | Bilateral |
| 67 | Dorsal raphe nucleus | DRaphe | Raphe Nuclei | Medial |
| 68 | Medial raphe nucleus | MRaphe | Raphe Nuclei | Medial |
| 69 | Cerebellum | Cereb | Cerebellum | Medial |

**2. Estimates of variability within cytoarchitectonic areas**

We estimated the accuracy of regional labels from the cytoarchitectonic template used in (Sawiak 2018) from (Majka P et al. 2016) and based on (Paxinos 2012). The recently published probabilistic maps (Majka 2021) show the correspondence of warped labels from a histological template to 20 individually labelled marmosets. It combined Nissl-stained sections to produce a three-dimensional image volume for each individual. Labels for each individual were produced using image co-registration from the Paxinos marmoset atlas (as originally used in Sawiak et al. 2018). Since the original data were histological, anatomical structures could be examined for validation of cytoarchitectonic labels. Using this approach, Majka et al. produced a probability map for each of 116 cortical areas highlighting how many of the 20 individual marmosets matched the template at every voxel. Resampling those maps to the 250µm resolution used in the current study, the mean probability values for the cortex within each areal mask is 75.6%.

Taking into account the resolution of the images used in our previous study (Sawiak 2018), we had already merged smaller cortical regions together (for example, regions A13L, A13M, A13a, A13b are considered as simply A13). Using the same labels for the histological dataset, gives a mean 79.2% probability of each voxel as belonging to a particular cytoarchitectural area. In terms of cortical variability, 92.1% of all voxels are more likely than not to be the label they have been given. Moreover, 40.4% of all cortical voxels are more than 90% likely to be the region they are labelled. As Majka et al. point out, most of the uncertainty concerns voxels on the boundary between two neighboring cortical regions. These are not problematic in the present study as the Jacobian determinant fields are locally smooth due to the regularization used in the image registration process (Ashburner J 2007). As MRI avoids the artefacts associated with sectioning and differential shrinkage during the staining processes in histology, it is likely that the individual variability is lower in the present dataset.

There is no such comprehensive histological dataset for young animals to date. However, we have already shown the most rapid period of growth occurs in early infancy with regional volumes (and total intracranial volume, TIV) peaking by approx. 6 months followed by a much slower reduction in volumes towards adulthood. In the present study, the mean reduction in overall brain size from adolescence to adulthood is 12%.

To show that an image registration approach to assigning cortical labels is reasonable, Figure S1 highlights brain changes between 12 months and adulthood. This was done by using the image registration transformations to warp our common template (comprising all adults and 12-month animals) to produce an average brain representative of each group. Each ‘mean brain’ representative of each group is calculated by averaging the displacement transforms at each point within the template across individuals within each cohort. Taking contours of the grey matter segmentation of each image enables an overview of the important morphological differences from 12 months to adulthood. This shows the overlaid contours from the two average brains, indicating the scale of change between them. A further set of contours were produced after the brains were adjusted with global scaling factors, indicating consistent overlap of important brain features once whole brain volume has been taken into account.

Both images were produced by registering the 12-month average brain to the adult average (SPM with 6 and 9 parameter coregistration, respectively).

The figure shows that the large-scale changes in brain morphology that occur in neonatal and very early infancy have largely concluded by 12 months and the remaining changes are well within what can reasonably be expected of an image registration process.


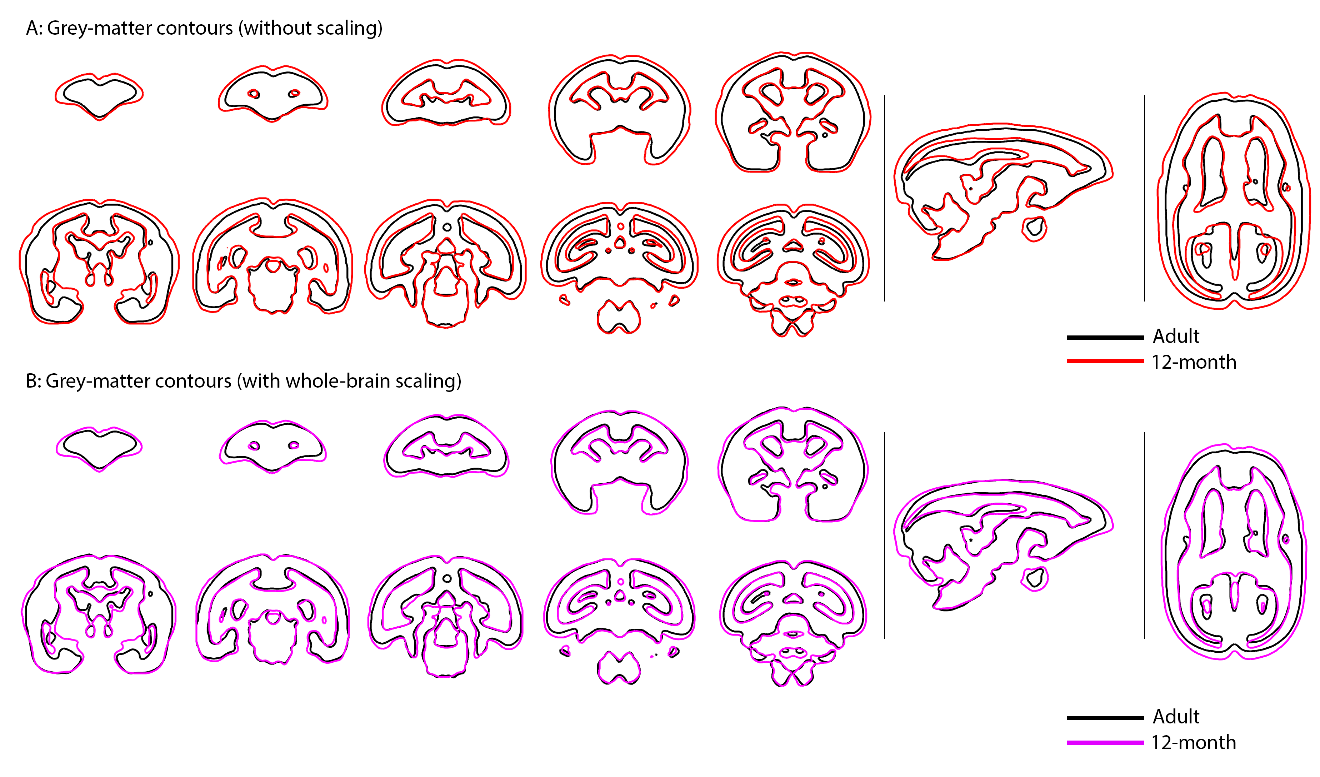


**Supplementary** **Figure 1:** **Contours showing shape differences between the mean 12-month and adult brains without correcting for overall brain volume (A) and with such corrections (B).** The data show that the brains from 12-month animals show similar gross morphology without substantial shape differences to those of adults, particularly when overall brain volume has been taken into account.

**3. Construction of maturational trajectory splines**

Data and methods from Sawiak et al. (2018) were used to construct splines representing marmoset volume growth trajectories from 3-24 months. The dataset is an accelerated longitudinal design from 41 animals scanned from 1-7 times each (a total of 147 scans, an average of 3.7 times per animal). In contrast to our earlier study, which had meaned volumes between hemispheres (creating trajectories for 69 areas), here we used individual splines for both left and right structures (the same 135 regions used in the structural covariance analysis).

In brief, volumes for each region were found by integrating Jacobian determinants from the image registration process and these were fitted with an additive mixed model with cubic splines. Specifically, the volume of each regional measurement by age and animal is given by a random effect for subject (time-invariant), a fixed effect for sex and a function of time per region.

The function of time was constructed from cubic b-splines (MATLAB 2019b, Mathworks). Here we used spline knots at 8-week intervals from 8-104 weeks (2-24 months). In our developmental study, we found useful milestones of development based not only on the splines but also their derivatives. The derivatives usefully highlight phases of growth and plateaus for different regions, and we therefore included them in creating our maturational trajectory covariance matrices. An illustration of the modelling process is shown in Figure S1, using left primary visual cortex (V1) as an example.

For each region, spline coefficients were normalized ($l^{2}$norm) independently for the splines and the derivatives, to remove overall size effects from the coefficients. This process leads to 135 sets of 13 volume trajectory coefficients and 13 derivative coefficients. Before concatenation, each set of 13×135 coefficients was standardized (as $z$-scores).


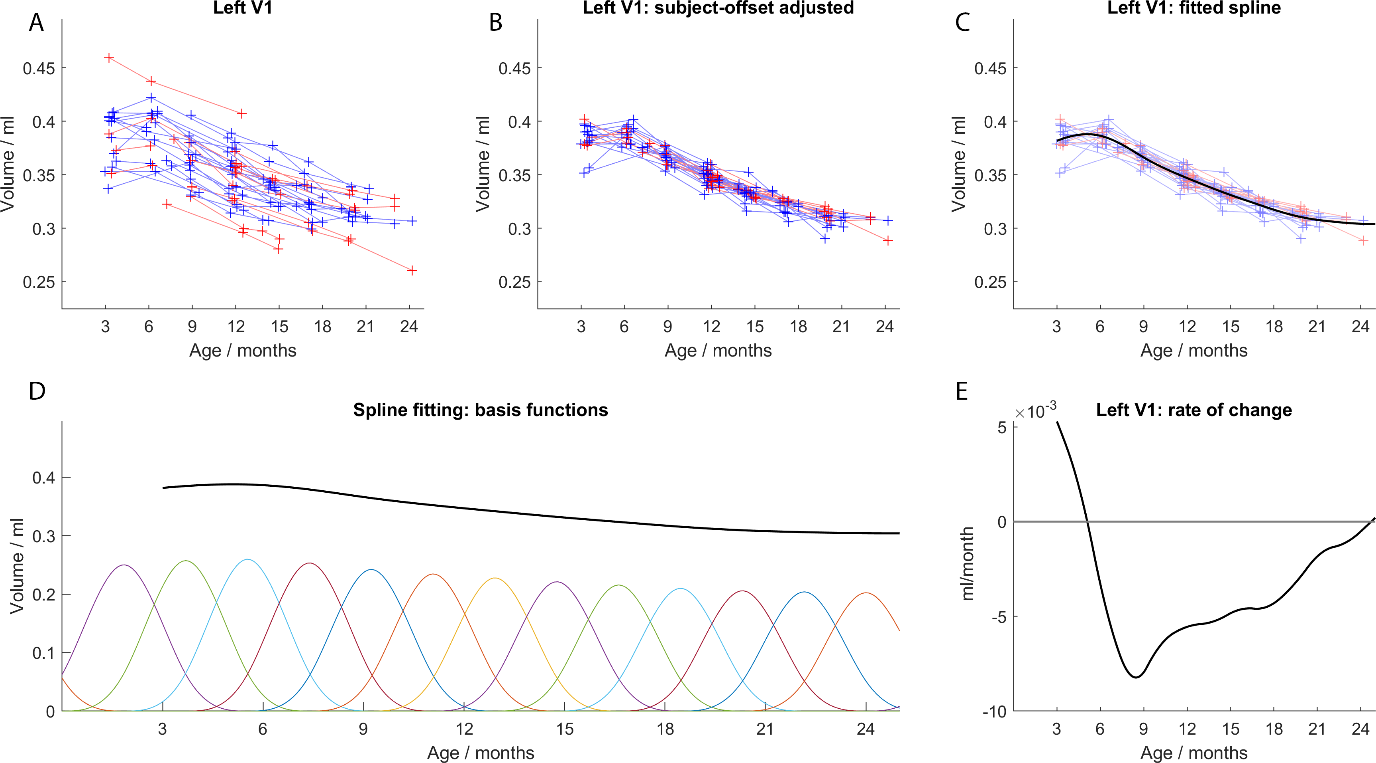


**Supplementary** **Figure 2: Spline coefficients of maturational trajectories.** (A) volume measurements for left V1 are shown for each animal (measurements from the same animals joined by lines; red/blue indicate female/male, respectively). (B) A subject offset is calculated as a time invariant mean volume shift per animal and the fitted spline is shown in (C). Although shown separately here, the subject offset and spline coefficients are calculated iteratively to find a least-squares solution of both at the same time. In (D) the same fitted spline is shown on a broader scale to show effect of each spline elements seen in (D). The ‘knots’ of each spline are their time center, and the effect of each coefficient (weight) is to adjust the volume locally at that time. The derivative of the spline curve is shown in (E) indicating the rate of change of volume at each time point (note for example it is zero at the maximum volume, approx. 5 months here.)

**4. Tract-tracing data vs. adult structural covariance networks**


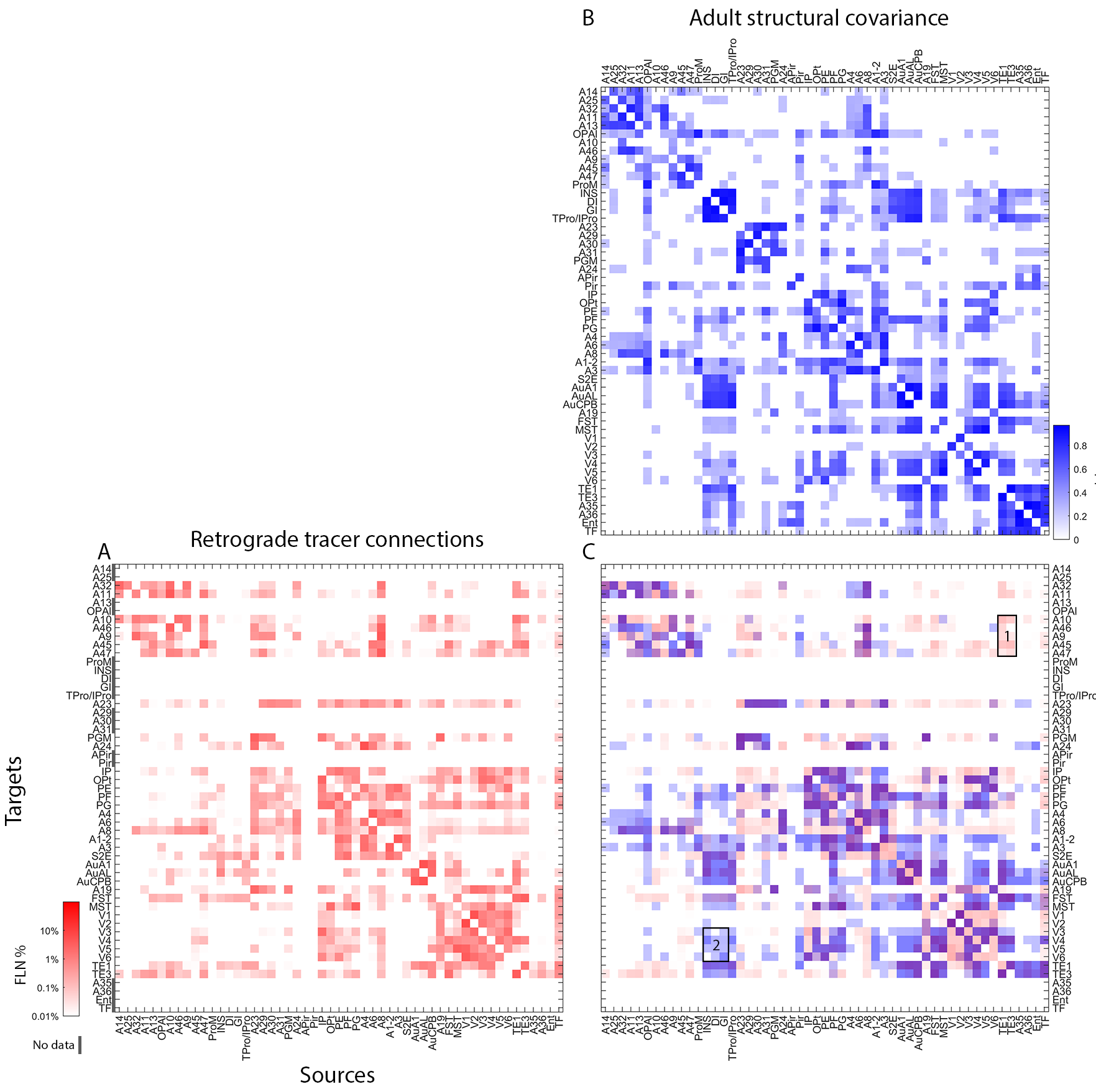


**Supplementary** **Figure 3:** **Comparison between a) tract-tracing data (Majka, 2020) and b) adult structural covariance network and c) their overlap**. Retrograde tracing data is shown for regions forming more than 0.01% of projecting source neurons to each target, and the adult structural covariance network has been thresholded at p(FDR) < 0.05. The tract-tracing dataset only includes 35 of the 53 cortical regions described in this study. Those regions not targeted by tract tracing are highlighted with a gray bar. The purple shading in c) reveals the considerable overlap between axonal connections and structural covariance between regions (rows without retrograde tracer targets have been blanked in (c) for clarity). Nevertheless, there are exceptions, whereby areas with many axonal connections show poor structural covariance (e.g., PFC areas with temporal cortex – box 1) and areas with few or no detected direct axonal connections still showing significant structural covariance (e.g., insula with visual cortex, box 2).

**References**

Ashburner J. 2007. A fast diffeomorphic image registration algorithm. Neuroimage 38:95-113

Sawiak SJ, Shiba Y, Oikonomidis L, Windle CP, Santangelo AM, Grydeland H, Cockcroft G, Bullmore ET, Roberts AC. 2018. Trajectories and Milestones of Cortical and Subcortical Development of the Marmoset Brain from Infancy to Adulthood. Cerebral Cortex. 28:4440–4453.

Majka P, Chaplin TA, Yu HH, Tolpygo A, Mitra PP, Wojcik DK, Rosa MG. 2016. Towards a comprehensive atlas of cortical connections in a primate brain: Mapping tracer injection studies of the common marmoset into a reference digital template. J Comp Neurol. 524:2161‐2181.

Majka P, Bai S, Bakola S, Bednarek S, Chan JM, Jermakow N, Passarelli L, Reser DH, Theodoni P, Worthy KH, Wang XJ, Wójcik DK, Mitra DP, Rosa MGP 2020. Open access resource for cellular-resolution analyses of corticocortical connectivity in the marmoset monkey. Nature Communications. 11:1–14.

Majka P, Bednarek S, Chan JM, Jermakow N, Liu C, Saworska G, Worthy KH, Silva AC, Wojcik DK, Rosa MGP. 2021. Histology-Based Average Template of the Marmoset Cortex With Probabilistic Localization of Cytoarchitectural Areas. Neuroimage. 226:117625.
